# Update on Sentinel Lymph Node Biopsy in Surgical Staging of Endometrial Carcinoma

**DOI:** 10.3390/jcm10143094

**Published:** 2021-07-13

**Authors:** Ane Gerda Z Eriksson, Ben Davidson, Pernille Bjerre Trent, Brynhildur Eyjólfsdóttir, Gunn Fallås Dahl, Yun Wang, Anne Cathrine Staff

**Affiliations:** 1 Department of Gynecologic Oncology, Oslo University Hospital, Norwegian Radium Hospital, N-0310 Oslo, Norway; uxbjpa@ous-hf.no (P.B.T.); bryeyj@ous-hf.no (B.E.); UXGUDA@ous-hf.no (G.F.D.); yunwang@ous-hf.no (Y.W.); 2 Institute of Clinical Medicine, Faculty of Medicine, University of Oslo, N-0316 Oslo, Norway; BDD@ous-hf.no (B.D.); UXNNAF@ous-hf.no (A.C.S.); 3 Department of Pathology, Oslo University Hospital, Norwegian Radium Hospital, N-0310 Oslo, Norway; 4 Division of Obstetrics and Gynaecology, Oslo University Hospital, Ullevål, N-0424 Oslo, Norway

**Keywords:** endometrial cancer, sentinel lymph node, lymphadenectomy

## Abstract

Sentinel lymph node (SLN) biopsy has emerged as an alternative staging approach in women with assumed early-stage endometrial carcinoma. Through image-guided surgery and pathologic ultrastaging, the SLN approach is introducing “precision medicine” to the surgical management of gynecologic cancers, providing a comprehensive evaluation of high-yield lymph nodes. This approach improves the surgeons’ ability to detect small-volume metastatic disease while reducing intraoperative and postoperative morbidity associated with lymphadenectomy. Although the majority of clinicians in Europe and the USA have recognized the value of SLN biopsy in endometrial carcinoma and introduced this as part of clinical practice, there is ongoing debate regarding its role in very low-risk patients as well as in patients at high risk of nodal metastasis. The significance of low-volume metastasis is not fully understood, and there is no consensus in regard to how the presence of isolated tumor cells should guide adjuvant therapy. Standardized protocols for histopathologic evaluation of SLNs are lacking. In this review article we aim to provide a framework for the introduction of SLN biopsy in endometrial cancer, give an updated overview of the existing literature, as well as discuss potential controversies and unanswered questions regarding this approach and future directions.

## 1. Introduction

Endometrial carcinoma is the most common gynecologic malignancy in Europe and North America. The estimated number of new cases worldwide in 2020 is 417,367, with an estimated 97,370 deaths [1]. There has been a steady increase in incidence over latter decades, in large part due to the global obesity pandemic and aging population. It is estimated that in 2040 there will be 608,100 new cases and 157,800 deaths, globally [1]. The majority of women with endometrial carcinoma present at an early stage, with disease confined to the uterus. It is well accepted that surgical staging should include hysterectomy and bilateral salpingo-oophorectomy (BSO). However, the role and extent of lymph node dissection is highly debated and without international consensus. Evidence suggests that lymphadenectomy provides prognostic information and directs the use of appropriate adjuvant treatment in patients who are node-positive. Furthermore, it eliminates the need for adjuvant treatment in low-risk patients with negative nodes and no extrauterine spread of disease. Since lymphadenectomy has not been associated with improved survival for women with apparent early-stage endometrial carcinoma [2,3], and carries risks of intra- and postoperative complications [4], sentinel lymph node (SLN) biopsy has emerged as an alternative staging approach thought to reduce potential complications associated with lymphadenectomy whilst still providing accurate staging. As SLN biopsy is gaining acceptance, there is debate regarding its role in patients with very low risk of nodal metastasis as well as in patients with a high risk. The prognostic role of very low-volume metastasis remains largely unknown, and there is a paucity of standardized histopathologic processing protocols for ultrastaging of SLNs. The purpose of this review article is to provide a background for the introduction of SLN biopsy in endometrial carcinoma, give an updated overview of the literature, and discuss potential controversies and unanswered questions regarding this approach.

## 2. Classification of Endometrial Carcinoma

The dualistic model described by Bokhman in 1983 characterized endometrial carcinomas as type I; stimulated by estrogen, typically preceded by endometrial hyperplasia, presenting at an early stage having a good prognosis, and type II; often developing in non-obese, elderly women, arising from an atrophic endometrium as serous or clear cell carcinoma [5,6]. This dualistic approach was very broad and lacked the ability to categorize tumors for accurate, targeted adjuvant therapy. In 2013 The Cancer Genome Atlas (TCGA) Research Network published the results of an integrated genomic, transcriptomic and proteomic characterization of endometrial carcinoma [7], identifying four distinct molecular subgroups correlating with progression-free survival. This study caused a paradigm shift in our understanding of endometrial carcinoma. Researchers in Vancouver and Leiden have since developed surrogate assays analogous to the TCGA molecular-based classification [8,9]. These molecular surrogate assays are integrated in the most recent ESGO/ESTRO/ESP guidelines for the management of patients with endometrial carcinoma, encouraging molecular classification in all patients, particularly in high-grade tumors [10]. Algorithms incorporating clinical, molecular and histopathologic risk factors with precision surgical staging, such as SLN mapping, should guide adjuvant therapy of women with endometrial carcinoma.

## 3. Rationale for Surgical Staging in Endometrial Carcinoma

The first International Federation of Gynecology and Obstetrics (FIGO) staging system of endometrial carcinoma was a two-stage system. Stage I was clinically confined to the uterus and stage II spread beyond the uterus. This staging system has since undergone multiple revisions, most importantly the expansion to a four-stage system in 1962, and a shift from clinical to surgico-pathologic staging in 1988, incorporating depth of myometrial invasion, identification of tumor cells in peritoneal cytology and metastasis to retroperitoneal lymph nodes [11]. This change in FIGO staging was based on findings published in the landmark study by Creasman describing surgical pathologic spread patterns of endometrial carcinoma and uterine factors increasing the risk of pelvic and para-aortic lymph node involvement [12], not on evidence demonstrating improved survival. Prior to this study, the treatment of endometrial carcinoma predominantly consisted of preoperative intrauterine radium placement by various techniques followed by hysterectomy and BSO. The shift to surgico-pathologic staging assumed that women would be treated by a primary surgical approach, and that histologic evaluation would guide adjuvant therapy.

Despite two randomized controlled trials demonstrating no survival benefit to lymphadenectomy in women with presumed early-stage endometrial carcinoma [2,3], there is lack of national and international consensus regarding the role and extent of nodal assessment in these patients. Although some retrospective studies have suggested that lymphadenectomy may have a therapeutic effect [13,14], a recent Cochrane review found no evidence that lymphadenectomy decreases risk of death or disease recurrence compared with no lymphadenectomy in women with presumed stage I disease [15].

Selective lymphadenectomy was evaluated by Mariani and colleagues in a prospective observational study applying the Mayo Clinic “low-risk” criteria. This study demonstrated that patients with the predefined low-risk features had less than a 1% risk of nodal metastasis, compared to a 16% risk in women who did not meet the low-risk criteria [16]. Low risk was defined as grade 1 or 2 endometrioid type with myometrial invasion (MI) ≤ 50% and primary tumor diameter ≤ 2 cm. This selective lymphadenectomy approach can prevent lymphadenectomy in low-risk patients, but will lead to unnecessary lymphadenectomy in many high-risk patients. The approach relies on intra-operative frozen section by experienced gynecologic pathologists, which is not readily available even in most major cancer centers.

## 4. The Sentinel Lymph Node Concept

### 4.1. History of Sentinel Lymph Node Concept

More than a century ago, the surgeons William Halsted and Herbert Snow hypothesized that cancer spread in an orderly fashion, initially to regional lymph nodes and thereafter to more distant sites. They subsequently advocated for lymph node dissection in patients with melanoma and breast cancer [17,18]. Sentinel node for carcinoma of the parotid gland was first described by Dr. Ernest Gould in 1960. Dr. Gould advocated that the sentinel node should be dissected and sent for frozen section and if found to be without malignancy a radical neck dissection could be omitted [19]. The first report of successful SLN mapping was in 1977 for lymphangiography of the penis and has since become standard of care in cutaneous melanoma, penile, breast and vulvar carcinoma [20]. The evolution of the initiation and acceptance of SLN mapping has been unique to each disease site, due to multiple variations in demographic and clinico-pathologic characteristics.

In gynecologic cancers, SLN mapping was first implemented in the setting of vulvar carcinoma. Vulvar tumors are easily accessible to injection of tracer, and the lymphatic drainage is well described following lymphatic channels to one or both groins. SLN biopsy in endometrial carcinoma was first reported by Burke and colleagues in 1996 [21]. In this pilot study, they injected isosulfan blue dye sub-serosally into the myometrium of 15 women with high-risk endometrial carcinoma and were able to identify uptake of dye in nodes in 67% (10 of 15) of cases. Several other observational studies followed, exploring injection sites and selection of dye, as well as oncologic and patient reported outcomes.

### 4.2. Sentinel Lymph Node Injection Site

Three main injection techniques have been evaluated for SLN mapping in endometrial carcinoma: cervical, hysteroscopic and laparoscopic fundal injection [22,23,24]. Cervical injection is preferable and has been adapted by the majority of surgeons due to its feasibility and high detection rates [25]. Some para-aortic lymph nodes may be reached only via the infundibulo-pelvic ligament pathway, which are not commonly accessible via the superficial cervical injection, and there is concern that some isolated para-aortic lymph node metastases are missed due to this. This is mainly of concern in patients with high grade and deeply invasive tumors, where isolated para-aortic metastasis has been reported in as many as 16% of patients [26]. These reports are from the pre-SLN era, prior to image-guided surgery and the introduction of pathologic ultrastaging and may thus have missed metastatic nodes in the pelvis. When retrospectively performing pathologic ultrastaging of patients with previously confirmed negative pelvic lymph nodes and isolated para-aortic metastasis, Multinu and colleagues found the prevalence of isolated para-aortic metastasis in their cohort to be reduced from 2.5% (10/394) to 1.8% (7/394) [27].

The Multicenter Italian Trials in Ovarian cancer (MITO) study group recently published the results from their prospective randomized controlled trial on hysteroscopic peritumoral injection versus cervical injection of indocyanine green (ICG) for sentinel node detection in endometrial carcinoma, where the primary endpoint was para-aortic detection rate [28]. The hysteroscopic injection route detected more para-aortic SLNs than the cervical injection route, however this difference did not reach statistical significance. The bilateral pelvic mapping rate was 60% in the hysteroscopic injection group versus 85% in the cervical injection group. This study supports the use of cervical injection with ICG for SLN biopsy in surgical staging of endometrial carcinoma. Surgeons should however consider the potential drawbacks of cervical injection on para-aortic SLN detection in women with higher risk of para-aortic metastasis.

### 4.3. Choice of Tracer and SLN Algorithm

The main goal of the SLN approach is to identify high-yield lymph nodes, limiting the need for comprehensive lymphadenectomy. To achieve this, the technique must have a high bilateral SLN detection rate, a high sensitivity for detection of metastatic lymph nodes and a low false negative rate. Multiple studies have evaluated the detection rates of SLNs in endometrial carcinoma using various tracers, alone and in combination. The use of ICG is associated with higher rates of bilateral SLN detection than blue dye. However, the combination of a radiotracer and blue dye is comparable to IGC alone and may be an option in centers where ICG and near infrared imaging is not available [29,30,31,32]. The randomized FILM (Fluorescence Imaging for Lymphatic Mapping) study randomized patients to SLN mapping with isosulfan blue followed by ICG or ICG followed by isosulfan blue [32]. This study demonstrated that ICG had a significantly higher overall and bilateral (96% vs. 74% and 78% vs. 31%, respectively) SLN detection rates than blue dye, and a post-hoc analysis showed that 38% of metastatic nodes would have been missed with the use of blue dye alone. ICG and near-infrared imaging is considered the gold standard for SLN detection in women with endometrial carcinoma.

Adherence to a SLN algorithm significantly improves the sensitivity and decreases the false negative rate of the SLN approach [33]. The accuracy of SLN biopsy to detect metastatic disease in endometrial carcinoma has been established in several prospective trials [34,35,36,37] (Table 1). The SENTI-ENDO trial was first to evaluate the accuracy of SLN biopsy using dual cervical injection of technetium and blue dye. The study showed an overall detection rate of 89% and a negative predictive value (NPV) of 97% [34]. These findings were affirmed in the FIRES (Fluorescent Imaging for Robotic Endometrial Cancer Sentinel lymph node biopsy) study, where women with clinical stage I endometrial carcinoma of all histologies and grades were included. A cervical injection of ICG with SLN biopsy followed by pelvic (340 cases) ± para-aortic (196 (55%) cases) lymphadenectomy, using the robotic platform. This study reported a sensitivity to detect node-positive disease of 97.2% and a NPV of 99.6% [35]. Most recently, the Sentinel Lymph Node Biopsy vs. Lymphadenectomy for Intermediate- and High-Grade Endometrial Cancer Staging (SENTOR) study evaluated the accuracy of SLN biopsy using cervical injection of ICG in intermediate- and high-grade endometrial carcinoma [37]. The SENTOR study demonstrated a sensitivity of 96%, negative predictive value of 99% and false negative rate of 4% when applying the SLN algorithm. These results are comparable to rates found in breast cancer and melanoma where SLN biopsy is standard of care, and established the accuracy of SLN biopsy in endometrial carcinoma, importantly also in women with high risk of nodal metastasis.

Of note, the above-mentioned prospective multicenter trials showed lower rates of bilateral mapping than single-center studies. This may reflect the importance and impact of case load and experience in injection technique as well as surgical technique for successful mapping and diagnostic accuracy. When implementing the SLN approach, individual surgeons should track their mapping rates and oncologic outcomes, particularly nodal recurrences in SLN negative cases. Failure of SLN mapping can be due to disruption of lymphatic channels or distorted anatomy due to previous surgery or pelvic radiation. Increasing body mass index (BMI) also decreases the rate of successful SLN mapping in women with endometrial carcinoma [38,39]. As per the Memorial Sloan Kettering Cancer Center (MSKCC) SLN surgical algorithm, any suspicious nodes should be removed, and a side-specific lymphadenectomy is performed in any unmapped hemi-pelvis [33]. Failure of SLN identification can be due to empty nodal packets; to avoid this the surgeon should palpate excised SLNs at time of surgery. In an attempt to standardize SLN dissection in endometrial carcinoma, Dr. Moloney and colleagues recently published their study on the development of a surgical competency assessment tool for sentinel lymph node dissection by minimally invasive surgery for endometrial carcinoma, applying a Delphi methodology including 35 expert gynecological oncology surgeons from 16 countries. The purpose of this study was to develop and validate a competency assessment tool for use in surgical quality assurance by identifying mandatory and prohibited steps of SLN dissection in endometrial carcinoma [40]. This tool can be helpful for surgeons and departments initiating a SLN mapping program to check for surgical proficiency as well as whether SLN dissection has been performed in accordance with an agreed standard.

### 4.4. Pathologic Ultrastaging and Low-Volume Metastasis

When introducing a SLN algorithm approach it is mandatory to include pathological ultrastaging of the harvested SLNs. SLN metastasis are reported according to the American Joint Committee on Cancer (AJCC) guidelines for the staging of breast cancer. Macrometastasis are defined as groups of malignant cells > 2.0 mm. Micrometastasis are defined as tumors within a lymph node measuring >0.2 mm and/or >200 cells, but ≤2.0 mm. Isolated tumor cell (ITC) clusters are small clusters of cells ≤ 0.2 mm, present either as single tumor cells or clusters of <200 cells [41]. The clinical and prognostic significance of low volume metastasis (ICTs) in endometrial carcinoma is largely unknown and a topic of debate [42]. The controversy regarding the value of ultrastaging in endometrial cancer may reflect the general uncertainty surrounding the value of lymphatic assessment in this disease.

There are currently no evidence-based guidelines for the pathologic assessment of SLNs in endometrial carcinoma. There is considerable variation between institutions with respect to the number of sections examined by routine Hematoxylin and Eosin (H&E) staining, depth of sectioning into tissue blocks, the interval of microns between parallel sections and the use of immunohistochemistry (IHC) to identify tumor cells not noted on H&E stain alone [43]. Some centers have adopted one-step nucleic acid amplification (OSNA) in evaluation of SLNs and showed that patients with metastatic non-SLNs had macrometastasis in the positive SLNs as defined by OSNA [44,45]. OSNA could potentially replace intraoperative frozen section of SLNs in cases where the status of metastatic vs. non-metastatic SLN would influence intraoperative management. This may be of greater clinical significance in patients with assumed early-stage cervical carcinoma than those with endometrial carcinoma. Further studies are needed to understand the potential role of OSNA in the detection of SLN metastasis in gynecologic cancers.

### 4.5. Oncologic Outcomes

Although the diagnostic accuracy of SLN mapping and biopsy is well-established, oncologic outcomes comparing SLN biopsy to comprehensive lymphadenectomy have not been investigated in prospective randomized trials, for any histologic subtype. It is generally accepted that SLN biopsy is sufficient for nodal assessment and without detriment to patients with negative SLNs due to the excellent NPV of this approach. Plante and colleagues explored the risk of metastasis in remaining non-SLNs in patients with a positive SLN, evaluating 268 women with apparent early-stage endometrial carcinoma [46]. They found that when the size of the SLN metastasis was ≤2 mm, the risk of having another positive lymph node was 5%, conversely, when the size of the SLN metastasis was >2 mm, the risk of having another positive lymph node was 60.8%. Histologic type, grade, depth of myometrial invasion, LVSI, cervical stromal invasion and CA-125 were not predictive. By this information, SLN biopsy should not influence oncologic outcomes significantly in women with SLN micrometastasis or ITCs. Although two randomized clinical trials have not demonstrated a survival benefit to lymphadenectomy in endometrial carcinoma [2,3], the therapeutic effect of removing metastatic nodes remains a controversial topic. There is ongoing debate, whether patients with high-risk endometrial carcinoma should undergo comprehensive lymphadenectomy, and by the same token if patients with positive SLNs should undergo completion lymphadenectomy. The Endometrial Cancer Lymphadenectomy Trial (ECLAT) started recruitment in March 2018 and may answer this question. The primary aim of this trial is to ascertain whether or not systematic pelvic and para-aortic lymphadenectomy have a significant impact on overall survival in patients with endometrial carcinoma FIGO Stages I or II and high risk of recurrence; this includes FIGO IB or II all histologic subtypes and FIGO IA endometrioid G3 or non-endometrioid endometrial carcinoma. In total, 640 patients will be randomized. In arm A, a total hysterectomy, bilateral salpingo-oophorectomy and in case of serous or clear cell histology an omentectomy will additionally be performed. In arm B, systematic pelvic and para-aortic lymphadenectomy up to the level of the left renal vein will additionally be performed. Final results from the ECLAT trial are not expected until 2029 [47].

Our knowledge regarding oncologic outcomes after SLN biopsy is limited to retrospective observational studies (Table 2). Historic cohorts from the Mayo Clinic and Memorial Sloan Kettering Cancer Center were compared to evaluate oncologic outcomes comparing SLN mapping and selective lymphadenectomy in women with endometrial carcinoma at low risk of nodal metastasis [48]. Of 1135 cases identified, 642 (57%) were managed with an SLN approach and 493 (43%) with a lymphadenectomy approach. Metastasis to pelvic LNs was detected in 5.1% and 2.6% of patients, respectively, and to para-aortic LNs in 0.8% and 1.0%, respectively. The three-year disease-free survival rates were 94.9% and 96.8% respectively, suggesting that both approaches are reasonable in detecting nodal metastasis with similar oncologic outcomes. When comparing oncologic outcomes in patients with deeply invasive endometrioid endometrial carcinoma there was no association between type of nodal assessment and recurrence or overall survival [49]. The same group compared oncologic outcomes after lymph node assessment by a SLN algorithm (118 cases) vs. comprehensive pelvic and para-aortic lymphadenectomy (96 cases) in patients with serous and clear cell endometrial carcinoma, and found that overall survival (OS) was not compromised with the SLN algorithm [50]. The study found that SLN may be associated with a decreased recurrence-free survival in this population, but similar OS in node-negative cases despite the majority receiving chemotherapy [50].

Schiavone and colleagues reported outcomes of 136 women with uterine carcinosarcoma [51]. In total, 48 patients underwent surgical staging with SLN mapping and 88 had routine lymphadenectomy consisting of pelvic and/or para-aortic lymph node dissection. There was no difference in median progression-free survival between the groups (23 vs. 23.2 months), and recurrence was distant/multifocal in 70% and 74% of patients in the SLN and lymphadenectomy groups, respectively. Another single-center study comparing SLN mapping to lymphadenectomy in patients with uterine serous carcinoma found no significant difference in two-year OS in stage I/II disease (96.6% vs. 89.6%), or in stage III disease (73.6% vs. 77.3% in the SLN and lymphadenectomy cohorts, respectively) [52]. The lack of consensus and standardization of adjuvant therapy in endometrial carcinoma may challenge interpretation of trials evaluating nodal approach as well as the design of future trials.

We await the results of two prospective trials evaluating oncologic outcomes: the ENDO 3 trial and the SELECT (Sentinel Lymph node Endometrial Cancer) Trial. ENDO 3 is a phase III randomized clinical trial comparing sentinel node biopsy with no retroperitoneal node dissection in apparent early-stage endometrial carcinoma. The study started in January 2021 and the expected completion date is January 2031. This trial aims to determine the value of SLN biopsy for patients, the healthcare system and to exclude detriment to patients. Its objectives are twofold; Firstly, to determine the recovery of participants (defined as incidence of adverse events, lower limb lymphedema and health-related quality of life) and to the healthcare system (cost) of SLN biopsy for the surgical treatment of endometrial carcinoma. Secondly, to compare disease-free survival at 4.5 years for participants randomized to receive hysterectomy, bilateral salpingo-oophorectomy with SLN biopsy compared to participants randomized to hysterectomy, bilateral salpingo-oophorectomy without retroperitoneal node dissection [53].

The SELECT trial, is a prospective multicenter international single-arm observational study on the oncological safety of the SLN algorithm in stage I intermediate-risk endometrial carcinoma. The study started in February 2020, expected primary completion date is February 2024. Its primary outcome measure is to assess the 36-month incidence of pelvic/non-vaginal recurrence in women with pathologically confirmed stage I intermediate-risk endometrioid endometrial carcinoma who have bilateral negative pelvic sentinel lymph nodes [54].

### 4.6. Patientreported Outcomes

Lymphadenectomy is thought to increase the risk of lower extremity lymphedema (LEL) in women having undergone surgery for endometrial carcinoma [4]. In addition to lymphadenectomy, these women commonly have comorbid conditions such as hypertension, diabetes and obesity, further increasing their risk of developing LEL. Women with chronic lymphedema often suffer leg heaviness, erythema, ulcers and pain requiring life-long treatment and psychosocial support [55]. Women report significant unmet needs related to pain or discomfort due to lymphedema [56]. One of the expected benefits of the sentinel lymph node approach is a reduction in LEL, however, there is limited data on this topic internationally. Validated questionnaires to detect LEL have been developed in relation to the GOG 244 study, designed to evaluate the incidence and risk factors for lymphedema associated with surgery for gynecologic malignancies [57,58,59], and by the Mayo group [60].

Leitao and colleagues investigated the prevalence of patient-reported LEL with SLN mapping versus comprehensive lymph node dissection, and found that SLN mapping was independently associated with a significantly lower prevalence of patient-reported LEL (27% vs 41%). They also found that high BMI and adjuvant external beam radiotherapy were associated with an increased prevalence of patient-reported LEL [61]. One challenge in studying LEL is the lack of standardized measurement techniques for LEL. Pigot and colleagues recently reported from a prospective, longitudinal gynecological cancer cohort study to determine LEL incidence up to 24 months post-diagnosis of endometrial carcinoma using bioimpedance spectroscopy (BIS) and self-reported leg swelling (SRLS) [62]. In this study the overall incidence of LEL was 33% and 45% according to BIS and SRLS, respectively. When analyses were restricted to obese women, incidence increased to 67% (BIS) and 54% (SRLS). Further prospective studies capturing quality of life and LEL in women with early-stage endometrial carcinoma are needed, as well as a standardization of measurements to refine and redefine the optimal risk-reduction strategies to diminish morbidity associated with treatment of gynecologic cancers [63].

## 5. Conclusions and Future Perspectives

Sentinel lymph node biopsy is increasingly used as an alternative to lymphadenectomy in surgical staging of women with endometrial carcinoma. The approach has gained significant acceptance and is applied in many centers. There is robust evidence regarding the accuracy of SLN biopsy for nodal staging in all risk-categories of endometrial carcinoma, however, prospective data on oncologic outcomes are lacking. The significance of low-volume disease identified by ultrastaging remains unknown. Future research should focus on understanding the optimal clinical management of this sub-group of patients. Standardized histopathological, and possibly molecular assessment protocols of SLNs is lacking. Reaching a consensus regarding histopathologic evaluation is important as the SLN approach is gaining acceptance and becoming more widespread. Oncologic outcomes, particularly in women at high risk of nodal metastasis, is lacking, and should be the focus of prospective trials. Any trial investigating the SLN approach should include patient reported outcomes.

## Figures and Tables

**Table 1:** Prospective trials determining diagnostic accuracy of SLN mapping.

Study	Study Population	*N*	Dye	Injection	SLN Mapping	Metastatic Nodes	Sensitivity	NPV
SENTI-ENDO Ballester [34]	All histologies	133 125 evaluable	Technetium + patent blue	Cervical	Bilateral: 62% Unilateral: 89%	16%	84%	97%
FIRES Rossi [35]	All histologies	385 340 evaluable	ICG	Cervical	Bilateral: 52% Unilateral: 86%	12%	97.2%	99.6%
SHREC Persson [36]	All histologies	275 257 evaluable	ICG	Cervical	Bilateral: 82% Reinjection: 95%	21%	100%	100%
SENTOR Cusimano [37]	All histologies	156	ICG	Cervical	Bilateral: 76% Unilateral:87.5%	17%	96%	99%

SLN, Sentinel lymph node; NPV, negative predictive value; ICG, indocyanine green.

**Table 2:** Oncologic outcomes of patients with endometrial carcinoma having undergone SLN mapping.

Study	Study Population	Nodal Assessment	*n*	Metastatic Nodes	*p*-Value	DFS	*p*-Value	OS	*p*-Value
Eriksson [48]	Endometroid Myoinvasion < 50%	SLN LND	642 493	5.1% pelvic 2.6% pelvic	0.03	94.9% 96.8% (3-year)	nr	97.4% 95.4% (3-year)	0.07
Schlappe [49]	Endometrioid Myoinvasion > 50%	SLN LND	82 94	33.3% pelvic 14.8% pelvic	0.005	78.7% 77.7% (3-year)	nr	91.8% 77.6% (3-year)	nr
Schlappe [50]	Serous & Clear Cell	SLN LND	118 96	22% pelvic 20% pelvic	0.83	69% 80% (3-year)	0.32	88% 77% (3-year)	0.06
Schiavone [51]	Carcinosarcoma	SLN LND	48 88	17.5% nr	na	23 mo 23.2 mo	0.7	nr	na
Basaran [52]	Serous Carcinoma	SLN LND	79 166	26.5% 29.5%	0.63	58.8% 64.9% (2-year)	0.48	89.1% 83.9% (2-year)	0.85

SLN, sentinel lymph node; LND, lymphadenectomy; DFS, disease-free survival; OS, overall survival; mo, months; na, not applicable; nr, not reported.

## Data Availability

Not applicable.
